# Analysis of Epigenetic Factors in Mouse Embryonic Neural Stem Cells Exposed to Hyperglycemia

**DOI:** 10.1371/journal.pone.0065945

**Published:** 2013-06-11

**Authors:** Sukanya Shyamasundar, Shweta P. Jadhav, Boon Huat Bay, Samuel Sam Wah Tay, S. Dinesh Kumar, Danny Rangasamy, S. Thameem Dheen

**Affiliations:** 1 Department of Anatomy, Yong Loo Lin School of Medicine, National University of Singapore, Singapore, Singapore; 2 Lee Kong Chian School of Medicine, Nanyang Technological University, Singapore, Singapore; 3 John Curtin School of Medical Research, The Australian National University, Canberra, Australia; Indian Institute of Toxicology Research, India

## Abstract

**Background:**

Maternal diabetes alters gene expression leading to neural tube defects (NTDs) in the developing brain. The mechanistic pathways that deregulate the gene expression remain unknown. It is hypothesized that exposure of neural stem cells (NSCs) to high glucose/hyperglycemia results in activation of epigenetic mechanisms which alter gene expression and cell fate during brain development.

**Methods and Findings:**

NSCs were isolated from normal pregnancy and streptozotocin induced-diabetic pregnancy and cultured in physiological glucose. In order to examine hyperglycemia induced epigenetic changes in NSCs, chromatin reorganization, global histone status at lysine 9 residue of histone H3 (acetylation and trimethylation) and global DNA methylation were examined and found to be altered by hyperglycemia. In NSCs, hyperglycemia increased the expression of Dcx (Doublecortin) and Pafah1b1 (Platelet activating factor acetyl hydrolase, isoform 1b, subunit 1) proteins concomitant with decreased expression of four microRNAs (mmu-miR-200a, mmu-miR-200b, mmu-miR-466a-3p and mmu-miR-466 d-3p) predicted to target these genes. Knockdown of specific microRNAs in NSCs resulted in increased expression of Dcx and Pafah1b1 proteins confirming target prediction and altered NSC fate by increasing the expression of neuronal and glial lineage markers.

**Conclusion/Interpretation:**

This study revealed that hyperglycemia alters the epigenetic mechanisms in NSCs, resulting in altered expression of some development control genes which may form the basis for the NTDs. Since epigenetic changes are reversible, they may be valuable therapeutic targets in order to improve fetal outcomes in diabetic pregnancy.

## Introduction

Diabetes during pregnancy is a well-known risk factor for congenital anomalies in various organ systems including the nervous system [1]–[3]. Preconceptual control of diabetes has shown to reduce the incidence of congenital abnormalities in diabetic mothers [4]. On the other hand a tight glycemic control after organogenesis and embryogenesis have begun may prove insufficient to prevent or reverse the congenital defects that have already occurred [5], [6].

Global gene expression profiles of developing brain in embryos of diabetic mice has shown differential expression of several genes that control various functions such as metabolism, cellular physiological process, cell cycle progression and cell migration during brain development [7]. Neural stem cells (NSCs) are self-renewing multipotent cells giving rise to neuronal and glial cells (astrocytes and oligodendrocytes) in the central nervous system [8]. It has been shown that maternal diabetes alters the expression of several genes involved in neurulation [9], [10], proliferation and cell fate specification [11] of NSCs which may form the basis for neural tube defects. In the developing brain, NSCs differentiate into neuronal and glial cell types in a sequential fashion. Cell fate specification of NSC is determined by extracellular signals, transcription factors and intracellular programmes such as the epigenetic regulation of gene expression [12]–[14]. Recently, microRNAs (miRNAs) have been shown to be essential in the post transcriptional control of NSC fate and regulation of gene expression [13], [15].

Dietary changes in methyl donors have shown to alter DNA and histone methylation [16]. Animal studies have revealed that a choline -deficient diet during pregnancy results in decreased methylation in genes that control brain development [17]. It is therefore evident that the fetal epigenetic mechanisms can be influenced by maternal nutrition or metabolic disturbances. These epigenetic changes that are acquired during embryogenesis may even have long lasting effects on the offspring postnatally [18], [19]. It has been shown that high glucose causes persistent alterations in gene expression in human aortic endothelial cells [20] and human monocytic cell line [21] through histone modifications. In addition, excess glucose can increase histone acetylation by increasing acetyl CoA in the nucleus [22] suggesting a link between maternal hyperglycemia and fetal epigenome.

We therefore hypothesize that exposure of NSCs to hyperglycemia results in activation of epigenetic mechanisms (chromatin modification, DNA methylation and miRNA mediated) which alter the expression of genes during brain development. To address this, we used NSCs isolated from the forebrain of non-malformed embryos of streptozotocin induced diabetic pregnancy and cultured those in medium containing physiological glucose (PG, 5 mmol/l) concentration to examine epigenetic reprogramming caused *in utero* by maternal hyperglycemia. First, we examined the histone and DNA methylation status of NSCs to determine hyperglycemia induced changes in them. In order to examine the effect of maternal diabetes on epigenetic regulation of genes involved in NSC fate specification we selected two genes, Doublecortin (*Dcx*), and Platelet activating acetyl hydrolase, isoform 1b, subunit 1 (*Pafah1b1*), which are involved in neurogenesis and neuronal migration. These genes were selected since their expression levels were found to be altered in developing brain of malformed embryos from diabetic pregnancy (in our previous cDNA microarray analysis) [7]. We identified four miRNAs that were predicted to target both Dcx and Pafah1b1 and determined if Dcx and Pafah1b1 were targets of these miRNAs by altering specific miRNA levels in NSCs. Finally, we examined the role of each miRNA in NSC fate by differentiating the cells after knockdown of specific miRNAs in NSCs. We show for the first time that hyperglycemia alters epigenetic mechanisms in NSCs which may form the basis for neural tube defects observed in diabetic pregnancy.

## Materials and Methods

### Ethics Statement

This study was approved by the National University of Singapore Institutional Animal Care and Use Committee (IACUC)(approval number 122\09) and all procedures were in accordance with its guidelines.

### Animals and Culture of NSCs

Diabetes was induced in healthy 6–8 weeks old female Swiss albino mice (Centre for animal resources, CARE, NUS) by a single intraperitoneal injection of Streptozotocin (STZ, 75 mg/kg body weight, Sigma-Aldrich, St. Louis, MO, USA) freshly prepared in 0.01 M citrate buffer pH 4.5. One week later, the blood sugar levels of the mice were tested using a blood glucose meter (Abbott’s laboratories, Illinois, USA) and mice with non-fasting glucose levels of >200 mg/dL were confirmed to be diabetic and selected for mating. Timed mating was done by placing 3–4 diabetic female mice with one age matched healthy male mice, in cages overnight. The day when the copulation plug was seen was counted as embryonic day 0.5 (E 0.5). Only embryos from pregnant mice with non-fasting glucose >300 mg/dL were used as experimental group. Age matched control pregnant mice were purchased from CARE, NUS. On E13.5, the embryos were collected by caesarean section of diabetic and control mice that were anesthetized with pentobarbital (150 mg/kg body weight). All procedures using laboratory animals were in accordance with the guidelines of Institutional Animal Care and Use Committee (IACUC), NUS.

Primary culture of NSCs was obtained from the telencephalon region of embryonic brains as described previously [11], [23]. Briefly, the tissue sections were subjected to mechanical dissociation in DMEM/F12 (1∶1, Invitrogen Life technologies, Carlsbad, CA, USA) and the cell suspension was filtered through a nylon mesh (70 µm, BD biosciences, MA, USA). The cells were plated at a concentration of 10–15 cells/µl [24], [25] in DMEM/F12 medium with 5 mM/L D-Glucose (PG) concentration and supplemented with insulin-transferrin-selenium supplements (Invitrogen), 20 ng/ml EGF (Sigma- Aldrich, St. Louis, MO, USA) and an antibiotic antimycotic solution (Sigma- Aldrich) in T-75 flasks (Corning Life sciences, Lowell, MA, USA). The cultures were incubated at 37°C/5% CO_2_ for 5 days after which the supernatant containing freely floating neurospheres was collected in 50 ml tubes (Greiner Bio-One GmBH, Germany) and centrifuged at 800 rpm/5 min. Harvested neurospheres were dissociated with TryPLE™ Select (Gibco, Life technologies, Carlsbad, CA, USA) and re-plated (50–70 cells/µl) for 3–4 days, during which new neurospheres formed (Fig. S1 A). Subsequently, a second passage was done (as previously) and cells were grown for 4–5 days. NSCs were grown for 12–14 days in total and passaged at least twice before they were used for experiments. NSCs were stained for expression of intermediate filamentous marker, Nestin (Fig. S1 B–E). All neurospheres from embryos of control and diabetic pregnancy expressed Nestin (Fig. S1 B,C) and all cells within a neurosphere showed immunoreactivity to Nestin (Fig. S1 D, E).

### Exposure of NSCs to High Glucose in vitro

30–50 neurospheres derived from E13.5 embryos of control pregnancy cultured in PG medium were transferred to 6 well plates containing 2 ml medium with high glucose concentration (HG, 40 mM/L D-glucose) and cultured for 48 h.

### Differentiation of Neurospheres

5–10 neurospheres derived from E13.5 embryos of control or diabetic pregnancy was plated per well of a 24 well plate containing poly-ornithine coated coverslips. Each well contained 500 µl of medium with PG concentration and 2% FBS (and no EGF) in order to induce differentiation. The NSCs were allowed to differentiate for 3 days or 6 days *in vitro* after which the percentages of Gfap, Map2 or Ng2 positive cells were estimated by immunostaining.

### Immunostaining

For immunostaining, the neurospheres or differentiated cells were fixed with ice cold 4% paraformaldehyde (PFA) for 30 min and the fixed cells were permeabilized with phosphate buffered saline (PBS) containing 0.1% Triton-X 100 (PBS-Tx) for 20 min. Subsequently, the cells were blocked with 5% normal goat serum for 30 min before incubation with the different primary antibodies. The primary antibodies used were rabbit anti-Gfap antibody (1∶500, Chemicon, Temecula, CA, USA) or rabbit anti-Map2 antibody (1∶500, Chemicon) or rabbit anti-Ng2 antibody (1∶200 Chemicon) or mouse anti-Nestin antibody (1∶500, Millipore, USA) overnight at 4°C. The next day, cells were incubated with Cy3-conjugated goat anti-rabbit IgG secondary antibody (1∶100, chemicon) or rabbit anti-mouse IgG secondary antibody (1∶100 Chemicon) for 1 h at room temperature. Finally, the nucleus was counterstained with DAPI and the coverslips were mounted on glass slides with fluorescent mounting medium (DAKO, USA). Images were captured with Olympus FV1000 confocal microscope. For estimating the percentage of Map2 or Gfap or Ng2 positive cells, confocal images from at least five random fields were captured for each sample. The number of positive cells (Map2 or Gfap or Ng2) was counted in each field and data was represented as percentage of positive cells (Map2 or Gfap or Ng2) relative to the total number of cells in that field.

### Electron Microscopy

NSCs from various groups were harvested by centrifugation (800 rpm/5 min/4°C) and washed twice with PBS, after which the cells were fixed (2% PFA, 3% glutaraldehyde in PBS) for 1 h at room temperature. The fixed cells were harvested by centrifugation, washed thrice with PBS and incubated in PBS solution overnight at 4°C. The cells were then post fixed in 1% OsO4, pH 7.4 for 2 h at room temperature after which they were washed with 0.1 M phosphate buffer (pH 7.4). The cells were then dehydrated through an ascending ethanol series at room temperature and infiltrated with 100% acetone: resin (1∶6) overnight at room temperature. After three changes in resin, the cells were embedded in fresh resin and polymerized at 55°C for 1 h. Sections cut were supported on grids (150 nm) and the slides were stained with uranyl acetate (10 min) and lead citrate (8 min) at room temperature. Images were captured using Bio Twin CM 120 (Philips) electron microscope.

### Histone Protein Extraction and Assays

Total histone proteins were isolated from NSCs from various groups using EpiQuik total histone extraction kit (Epigentek, Farmingdale, NY) following the manufacturers’ instruction. Histone extracts (2 µg) from each sample was used to quantify Histone H3K9 trimethylation or acetylation using EpiQuik Global trimethyl histone H3K9 quantification kit (Epigentek, Farmingdale, NY) or EpiQuik Global acetyl histone H3K9 quantification kit (Epigentek) following the manufacturers’ protocol.

### DNA Isolation

Genomic DNA was extracted from NSCs from various groups using DNeasy blood and tissue kit (Qiagen, Hilden, Germany). The extracted DNA was quantitated using Nanodrop spectrophotometer.

### Global DNA Methylation Quantification

Genomic DNA (200 ng) from each NSC group was used to quantify the global DNA methylation levels using Methylamp global DNA methylation quantification kit (Epigentek, Farmingdale, NY) and the manufacturer’s instructions were followed. All samples were analyzed in duplicate. A standard curve was plotted using the methylated DNA control supplied in the kit and the DNA methylation % was calculated based on the standard curve.

### RNA Isolation

Total RNA was extracted from the control and experimental groups using mirVana™ kit (Ambion, Carlsbard, CA, USA) according to the manufacturer’s protocol. The isolated RNA was used for miRNA analysis or mRNA qRT-PCR.

### cDNA Synthesis and mRNA Analysis

Reverse transcription was done using 2 µg RNA and 2 µl oligodT, 200 U of molony murine leukemia virus (M-MLV) reverse transcriptase, 5 U of RNasin (Promega, Madison, WI, USA), 2 mmol/L of each dNTPs, in a 25 µl reaction volume. The mRNA expression was quantified by real time RT-PCR analysis carried out in Applied Biosystems (Applied Biosystems, Foster city, CA, USA) 7900 HT instrument using 10 µl master mix containing 5 µl SYBR green (Qiagen, Hilden, Germany), 1 µM/L of each primer (Table 1), 1 µl cDNA and Nuclease free water in 96 well FAST optical plates. The fold change of mRNA expression was calculated by 2^−ΔΔCt^ method [26].

**Table 1:** Primers used for mRNA qRT-PCR.

Gene name	Forward Primer(5′ –3′)	Reverse primer(5′- 3′)	Product size
***Dcx***	TCCAGTCAGCAAAGGTAAGGA	CCAAGAGAGAACAGCAAACCA	146 bp
***Pafah1b1***	GATGACAAGACCCTCCGTGT	GAGCTCAAATGGGGTAACCA	240 bp
***Map2***	CTGGACATCAGCCTCACTCA	AATAGGTGCCCTGTGACCTG	164 bp
***Gfap***	AGAAAACCGCATCACCATTC	TCACATCACCACGTCCTTGT	184 bp
***Ng2***	GCACGATGACTCTGAGACCA	AGCATCGCTGAAGGCTACAT	223 bp
***Beta actin***	GAAGAGCTATGAGCTGCCTGA	GGATTCCATACCCAAGAAGGA	103 bp

### Protein Isolation and Western Blotting

Protein was extracted from the NSCs using the mammalian protein extraction reagent (M-PER, Thermo scientific, Rockford, IL USA) following the manufacturer’s protocol. The extracted protein was quantitated using the Bradford method (Bio-Rad, Hercules, CA, USA). 20 µg of protein from each sample was denatured at 95°C for 5 min and separated on a 10% SDS-PAGE. The proteins were transferred to PVDF membranes and blocked with 5% non-fat milk for 1 h at room temperature. The blots were incubated in primary antibody, rabbit anti-Dcx antibody (1∶1000, Abcam, Cambridge, MA) or rabbit anti-Pafah1b1 antibody (1∶1000, Abcam, Cambridge, MA) or mouse anti-beta actin antibody (1∶5000, Sigma, St. Loius, MO,USA) overnight at 4°C. The blots were then incubated with secondary anti-mouse or anti-rabbit HRP conjugated antibody (Pierce, Rockford, IL, USA) for 1 h at room temperature. The blots were developed with enhanced chemiluminescence reagent (Pierce, Rockford, IL, USA) and quantitated on densitometer (Bio-Rad, Hercules, CA, USA) using Quantity One software (Bio-rad, Hercules, CA, USA). Equal protein loading was confirmed by stripping and re-probing the blots with beta actin antibody.

### Bisulphite Sequencing and Cloning

DNA (1 µg) from each NSC group was bisulfite treated and purified using Epitect bisulfite kit (Qiagen, Hilden, Germany), following the manufacturer’s protocol. Bisulfite specific primers were designed using Methylprimer Express softwareV1.0 (Applied Biosystems, Foster City, CA, USA) spanning 350–400 bp of CpG islands in the gene promoter. The bisulfite treated DNA was then amplified by PCR and the PCR amplicons were purified and subcloned into the TOPO TA^R^ cloning kit (Invitrogen, Life technologies, Carlsbad, CA, USA). Plasmid DNA was isolated from 6 positive colonies and sequenced. The eletropherograms were analyzed using the BiQ analyzer software (Max-Planck Institut fur Informatik, Germany) and the methylation pattern was represented as lollipop grids. Open circles represent unmethylated CpG sites and closed (shaded) circles represent methylated CpG sites. The primer sequences used for PCR and sequencing are listed in Table S1 in Tables S1.

### miRNA Target Prediction

We used the miRWalk database (http://www.ma.uni-heidelberg.de/appa/zmf/mirwalk/) to predict miRNA-mRNA interactions in the 3′UTR of the genes under study. The results of the search are summarized in Table 2.

**Table 2:** miRNA and mRNA target prediction by miRWalk.

mRNA	miRNA	No. of databases predicting interaction	Names of databases
***Dcx***	mmu-miR-200a	6	DIANAmT, miRanda, miRDB, miRWalk, PITA, Targetscan
	mmu-miR-200b	6	DIANAmT, miRanda, miRWalk, PITA, Targetscan, PICTAR 4
	mmu-miR-466a-3p	4	miRanda, miRWalk, PITA, Targetscan
	mmu-miR-466d-3p	4	miRanda, miRWalk, PITA, Targetscan
***Pafah1b1***	mmu-miR-200a	4	DIANAmT, miRanda, PITA, Targetscan
	mmu-miR-200b	5	.DIANAmT, miRanda, miRWalk, PITA, Targetscan
	mmu-miR-466a-3p	4	miRanda, miRWalk, PITA, Targetscan
	mmu-miR-466d-3p	4	miRanda, miRWalk, PITA, Targetscan

### In situ Hybridization

NSCs from normal pregnancy were transferred into 24 well plates with poly lysine coated coverslips and the cells were allowed to adhere for 48 h. 5′ Fluorescein labelled miRCURY LNA ™ probes were purchased for mouse U6, mmu-miR-200b and mmu-miR-466d-3p from Exiqon (Vedbaek, Denmark)(Table S2 in Tables S1). We followed a previously published protocol for *in situ* hybridization [27]. Briefly, the cells were fixed with 4% PFA, permeabilized with 0.1% PBS-Tx, and acetylated with acetylation solution containing acetic anhydride and triethanolamine. The probes were denatured and then allowed to hybridize to cells overnight at a temperature ranging from 55°C to 60°C (that was 22°C less than the melting temperature of the probe). Post hybridization washes were done at 75°C to 80°C (that was 20°C greater than hybridization temperature). The NSCs were counterstained with DAPI (1 µg/ml, Molecular probes) and coverslips were mounted on glass slides with fluorescent mounting medium (DAKO). Images were captured with Olympus FV1000 confocal microscope.

### miRNA Analysis

Mouse miRNA primers mmu-miR-200a, mmu-miR-200b, mmu-miR-466a-3p mmu-miR- 466d-3p, were purchased from Applied Biosystems Foster City, CA, USA (Taqman ™ miRNA Real time assay) and control primer set snoRNA234 was used to normalize the samples. Total RNA was reverse transcribed using miRNA specific stem-loop RT primers and subsequently the expression of miRNA was detected using specific miRNA primers. Mouse mmu-miR-124 and control U6 primers were purchased from Exiqon (Vedbaek, Denmark). Total RNA was converted to cDNA using the Universal cDNA synthesis kit (Exiqon, Vedbaek, Denmark) and the cDNA was used for quantification of miRNA-124.The miRNA expression was quantified by real time RT-PCR analysis using 96 well FAST optical plates (7900 HT, Applied Biosystems). The fold change in miRNA expression was calculated by 2^−ΔΔCt^ method [26].

### miRNA Knock Down

NSCs from normal pregnancy cultured in medium containing PG concentration were used for miRNA knockdown. Just before transfection, the NSCs were trypsinised gently to yield single cells and 2×10^5^ cells were seeded per well of a 24 well plate. Transfection was done using X-tremeGENE siRNA transfection reagent (Roche Applied Sciences, Mannheim, Germany) following the manufacturer’s instruction. 5′flourescently labelled miRCURY LNA ™ miRNA inhibitors mmu-miR-200b, mmu-miR-466d-3p, and non labeled miRCURY LNA ™ mmu-miR-200a, mmu-miR-466a-3p and were purchased from Exiqon (Vedbaek, Denmark) (Table S3 in Tables S1). Transfection complexes were prepared in opti-MEM medium (Invitrogen, Life technologies, Carlsbad, CA, USA) and added to the cells at final concentration of 20 nM/L. 5′ fluorescently labeled scrambled probe was used as the negative control. 48 h post transfection, protein expression of the predicted mRNA targets was analyzed by Western blot.

### miRNA Knockdown and Lineage Specification of NSCs

For lineage specification, the NSCs were harvested by centrifugation 48 h after knockdown with miRNA inhibitors and trypsinised to yield single cells. About 10,000 cells from scrambled or miRNA knockdown wells were plated in triplicate (one each for Gfap, Map2 and Ng2) in 24 well plates containing poly-ornithine (Sigma-Aldrich, St. Louis, MO, USA) coated coverslips. In order to induce differentiation, EGF was withdrawn from the culture medium and 2% FBS was added to the medium and cells were cultured for 24 h before proceeding with immunostaining using glial and neuronal cell lineage markers. For estimating percentage of cells, confocal images from at least five random fields were captured for each slide. The percentage of Gfap, Ng2 and Map2 positive cells were calculated in miRNA knockdown cells and normalized to scrambled transfected cells.

### Statistical Analysis

Data is represented as mean ± SD from at least three independent experiments. Student’s *t* test was done by using Microsoft Excel spreadsheet and data was considered significant when *p*<0.05.

## Results

### Chromatin Reorganization in NSCs Exposed to Hyperglycemia

**Figure 1:**
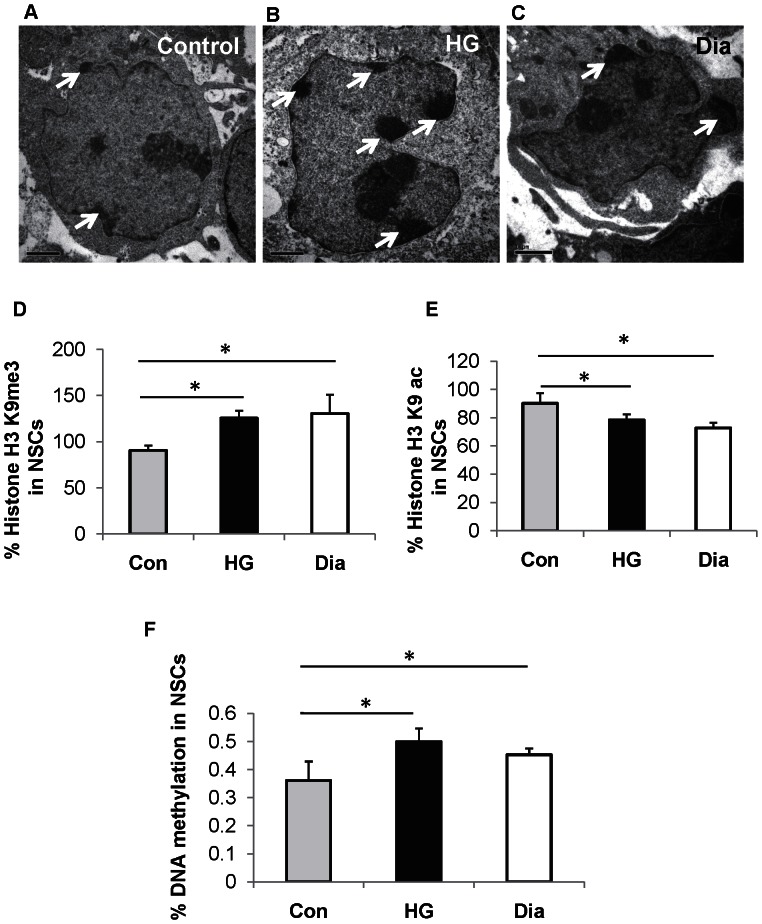
Hyperglycemia alters epigenetic mechanisms in NSCs. (A–C) Representative transmission electron microscopy images of NSCs from control (A), that exposed to high glucose (HG) *in vitro* (B) and from diabetic pregnancy (C). Increased heterochromatin structure at the nuclear periphery was observed in NSCs exposed to HG and that from diabetic pregnancy (white arrows). Bar represents 1 µm. (magnification: 13,500). (D, E) The percentage of H3K9me3 (D) was significantly increased in NSCs exposed to HG *in vitro* (black bars) and that from diabetic pregnancy (open bars) when compared to the control (grey bars) while the percentage of H3K9ac (E) was significantly decreased in NSCs exposed to HG *in vitro* (black bars) and that from diabetic pregnancy (open bars) when compared to the control (grey bars). (F) Increased percentage of DNA methylation was observed in NSCs exposed to HG *in vitro* (black bars) and that from diabetic pregnancy (open bars) when compared to the control (grey bars). The bar represents the mean ± SD of at least 4 independent experiments, *p<0.05.

In this study, NSCs were isolated from embryos of normal pregnancy and diabetic pregnancy and cultured in medium with PG. In addition, NSCs derived from embryos of normal pregnancy were cultured in medium with high glucose (HG) for 48 h so as to serve as *in vitro* model of diabetic pregnancy. The effect of hyperglycemia on chromatin organization in nuclei of NSCs was analyzed using transmission electron microscopy as it was hypothesized that hyperglycemia could trigger chromatin modifications in NSCs. Heterochromatin clumps were found to be increased (indicated as dark, dense regions) at the nuclear periphery of NSCs from diabetic pregnancy (Fig. 1C) and that exposed to HG *in vitro* (Fig. 1B) when compared to the control (Fig. 1A) suggesting that hyperglycemia triggers the reorganization of chromatin in embryonic NSCs. This also provides evidence that the chromatin reorganization observed in NSCs from embryos of diabetic pregnancy was indeed the effect of hyperglycemia as similar results were obtained when NSCs (derived from normal pregnancy) were cultured in HG *in vitro.*

### Increased Global Histone H3K9 Trimethylation and DNA Methylation and Decreased Histone H3K9 Acetylation in NSCs Exposed to Hyperglycemia

The chromatin consists of DNA that can be methylated and histone proteins that undergo modifications which control chromatin packaging and organization [28]. Hence we postulated that the chromatin reorganization observed in NSCs exposed to hyperglycemia is associated with histone modifications or DNA modifications. Therefore we analyzed the status of histone H3 lysine 9 (trimethylation and acetylation) and DNA methylation levels in NSCs. The status of histone modifications was examined by an ELISA based approach using two histone antibodies: anti-trimethyl histone H3 lysine 9 (H3K9me3) and anti-acetyl histone H3 lysine 9 (H3K9ac). The reason for selection of these two antibodies was because H3K9me3 is generally associated with transcriptional silencing, whereas H3K9ac is associated with transcriptional activation [29]. Interestingly, NSCs from diabetic pregnancy (130.42±20.41%, *p*<0.05) and that exposed to HG *in vitro* (125.48±7.98%, *p*<0.05) showed a significant increase in transcriptional silencing, when compared to the control (90.60±5.01%)(Fig. 1D). In addition, NSCs from diabetic pregnancy (72.63±3.79%, p<0.05) and that exposed to HG *in vitro* (78.34±4.05%, *p*<0.05) showed a significant decrease in transcriptional activation when compared to the control (90.13±7.31%)(Fig. 1E). Further, we examined the effect of hyperglycemia on global DNA methylation using an ELISA based approach. We found that the percentage of DNA methylation in NSCs from diabetic pregnancy (0.45±0.02%, *p*<0.05) and that exposed to HG *in vitro* (0.49±0.04%, *p*<0.05) was significantly increased when compared to the control (0.36±0.06%) (Fig. 1F).

### Epigenetic Regulation of Genes Involved in Neurogenesis, Neuronal Migration in NSCs Exposed to Hyperglycemia

**Figure 2:**
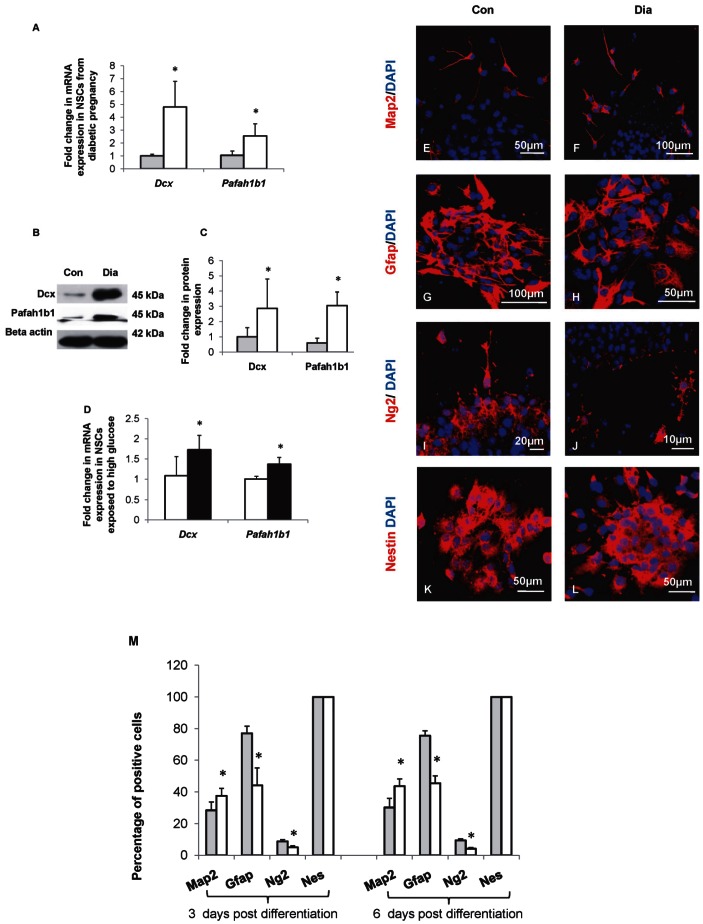
Increased Dcx and Pafah1b1 expression and increased neurogenesis in NSCs from diabetic pregnancy. (A) mRNA expression of *Dcx* and *Pafah1b1* increased significantly in NSCs from diabetic pregnancy (open bars) compared to the control (filled bars). (B, C) The expression and quantities of Dcx and Pafah1b1 proteins in NSCs from control and diabetic pregnancy were estimated by Western blot. (B) Representative blot shows the expression of Dcx and Pafah1b1 proteins in NSCs from embryos of control and diabetic pregnancy. (C) The quantities of Dcx and Pafah1b1 proteins increased significantly in NSCs from embryos of diabetic pregnancy (open bars) compared to the control (filled bars). (D) Dcx and Pafah1b1 mRNA increased significantly in NSCs exposed to HG *in vitro* (filled bars) when compared to the control (open bars). (E–L) Neurospheres from embryos of control and diabetic pregnancy were allowed to differentiate for 3 days *in vitro* and the expression of neuronal (Map2), glial (Gfap, Ng2), and Nestin positive cell populations were determined by immunocytochemistry. (M) The percentage of Map2 positive cells was significantly increased while the percentages of Gfap and Ng2 positive cells were significantly reduced in NSCs from diabetic pregnancy (open bars) on both 3 days or 6 days post differentiation when compared to the control(closed bars). Data is represented as mean ± SD from at least four independent experiments, *p<0.05.

We have previously demonstrated that maternal diabetes perturbed neurogenesis and neuronal migration in the developing neural tube resulting in NTDs [7]. To examine if the altered gene expression was due to changes in epigenetic mechanisms, we selected two genes namely, Dcx and Pafah1b1 that are involved in neurogenesis and neuronal migration. The expression of Dcx and Pafah1b1 mRNA and protein in NSCs from control and diabetic pregnancy were analyzed by qRT-PCR (Fig. 2A) and Western blot (Figs. 2B,C). The expression of *Dcx* (4.79±1.99 vs 1.00±0.13-folds, *p*<0.05) and *Pafah1b1* (2.55±0.93 vs 1.04±0.33-folds, *p*<0.05) mRNAs were significantly increased in NSCs from diabetic pregnancy when compared to the control (Fig. 2A). In addition, the quantities of Dcx (2.86±1.93 vs 1.00±0.60-folds, *p*<0.05) and Pafah1b1 (3.05±0.88 vs 1.00±0.51-folds, *p*<0.05) proteins increased significantly in NSCs from diabetic pregnancy when compared to the control (Fig. 2C). Further, the mRNA expression of Dcx (1.73±0.35 vs 1.08±0.47-folds, *p*<0.05) and Pafah1b1 (1.36±0.16 vs 1.00±0.06-folds, *p*<0.05) increased significantly in NSCs exposed to HG *in vitro* when compared to the control (Fig. 2D). In order to identify if the changes in mRNA expression in NSCs exposed to hyperglycemia (*in vivo* and *in vitro*) were due to changes in CpG methylation, we performed bisulphite conversion followed by cloning and sequencing of isolated DNA. There was no change in CpG methylation at Pafah1b1 gene promoter in NSCs from diabetic pregnancy or that exposed to HG *in vitro* when compared to the control (Fig. S2). CpG islands were found to be absent in the promoter of Dcx gene.

Subsequently, the percentages of neuronal and glial cells were estimated since hyperglycemia increased the expression of Dcx and Pafah1b1. The neurospheres of control and diabetic pregnancy were allowed to differentiate in medium with PG and 2% FBS, without EGF for 3 days or 6 days *in vitro* and the neuronal and glial populations were identified by immunostaining with Map2 (Microtubule associated protein 2) or Gfap (Glial fibrillary acidic protein) or Ng2 (neuron-glial antigen 2) or Nestin (Fig. 2E–L and Fig. S3 A–H) and quantified. The percentages of neuronal and glial positive cells were estimated after 3 and 6 days of differentiation in order to check if prolonged differentiation would alter the neurogenesis: gliogenesis ratio. All the differentiated cells from NSCs of control and diabetic pregnancy showed immunoreactivity to Nestin (Fig. 2 K,L, and Fig. S3 G,H). The percentage of Map2 positive cells increased significantly at 3 days (37.55±4.64 vs 28.41±5.30%, *p*<0.05) and 6 days (43.63±4.57 vs 30.17±5.71%, *p*<0.05) post differentiation. However, the percentages of Gfap positive cells at 3 days (44.19±10.97 vs 77.04±4.53%, *p*<0.05) and 6 days (45.47±4.72 vs 75.52±3.10%, *p*<0.05) and Ng2 positive cells at 3 days (5.05±1.00 vs 8.80±1.08%, *p*<0.05) and 6 days (4.14±0.73 vs 9.43±0.96%, *p*<0.05) decreased significantly in differentiated cells from NSCs of diabetic pregnancy when compared to the control signifying that hyperglycemia increased neurogenesis and decreased gliogenesis (Fig. 2M).

### Hyperglycemia Alters the Expression of miRNAs in NSCs

**Figure 3:**
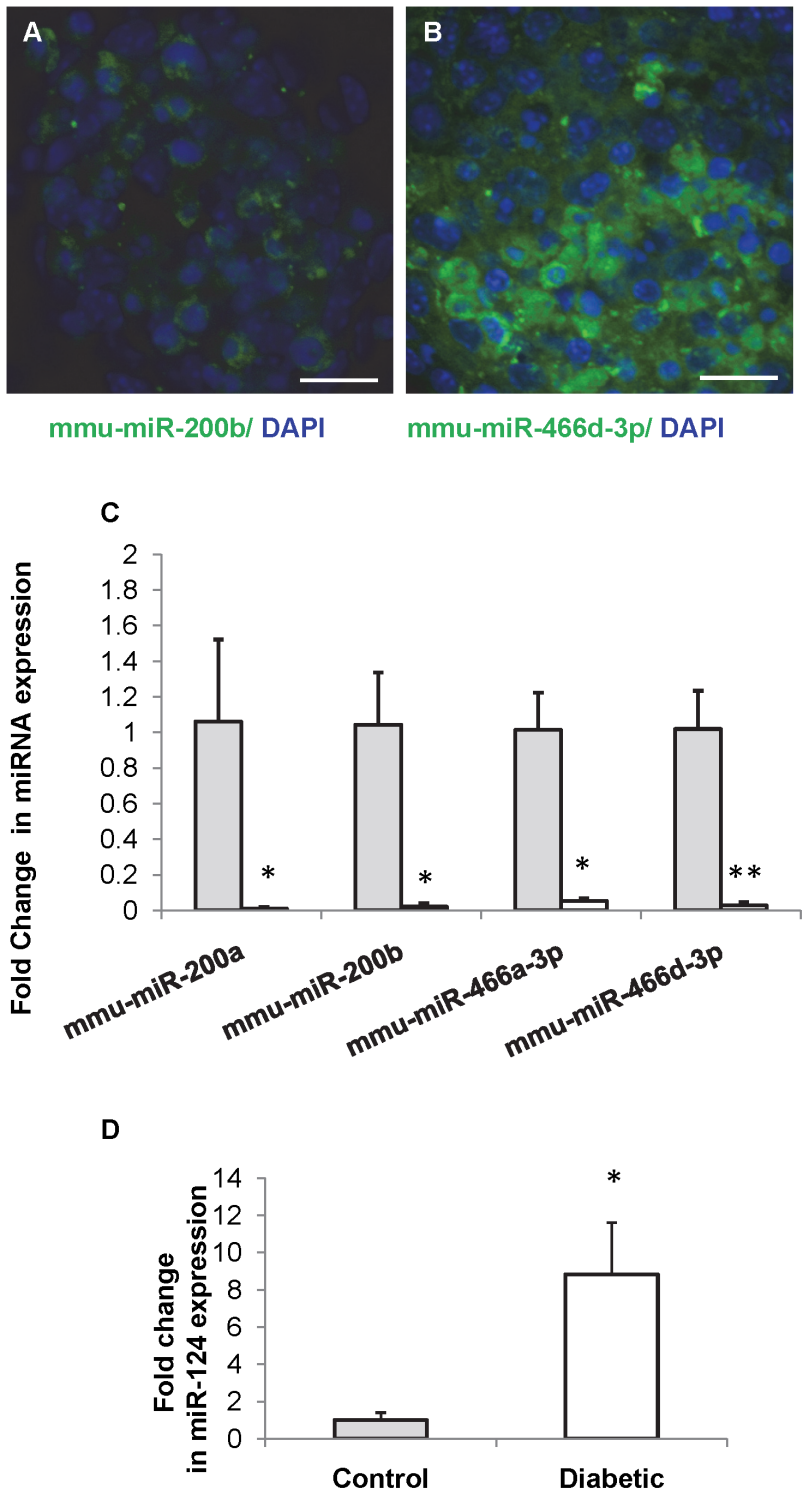
miRNA expression is altered in NSCs from diabetic pregnancy. (A, B) *in situ* hybridization reveals expression of miRNAs mmu-miR-200b (A) and mmu-miR-466d-3p (B) in NSCs. The nucleus is counterstained with DAPI (blue). Scale bar: 50 µm. (C) miRNA expression analysis revealed decreased expression of miRNAs mmu-miR-200a, mmu-miR-200b, mmu-miR-466a-3p and mmu-miR-466d-3p in NSCs from diabetic pregnancy (open bars) when compared to the control (filled bars). Mean ± SD (n = 4), *p<0.05, **p<0.01 (D) Expression of neuron specific miRNA, miR-124 was significantly increased in NSCs from diabetic pregnancy (open bars) when compared to the control (filled bars). Data is represented as mean ± SD from three independent experiments, *p<0.05.

miRNAs predicted to target the 3′ UTR of selected genes *(Dcx* and *Pafah1b1)* were identified from miRWalk database [30]. Among several miRNAs predicted to target proposed genes, we analyzed four miRNAs from two families (Table 2) that were common targets to selected genes. We performed *in situ* hybridization of two representative miRNAs (mmu-miR-200b and mmu-miR-466d-3p) selected from each family of miRNA that are investigated in this study (mmu-miR-200 and mmu-miR-466 family) in unbiased manner to detect whether these miRNAs were expressed by NSCs. Both mmu-miR-200b and mmu-miR-466d-3p were found to be expressed by NSCs (Figs. 3A,B). Having confirmed that these miRNAs were expressed in NSCs we then sought to quantitate their expression levels by real time RT-PCR in NSCs. The expression levels of miRNA mmu-miR-200a (0.009±0.009 vs 1.06±0.45-folds, *p*<0.05), mmu-miR-200b (0.021±0.02 vs 1.04±0.37-folds, *p*<0.05), mmu-miR-466a-3p (0.054±0.01 vs1.01±0.21-folds, *p*<0.05) and mmu-miR-466d-3p (0.028±0.02 vs 1.01±0.21-folds, *p*<0.01) were significantly decreased in NSCs from diabetic pregnancy when compared to the control (Fig. 3C). In NSCs from diabetic pregnancy, the significant increase in protein expression of Dcx and Pafah1b1 correlates well with the reduced expression of miRNAs (mmu-miR-200a, mmu-miR-200b, mmu-miR-466a-3p and mmu-miR-466d-3p) (Figs. 2B and 3C) which have been predicted to target Dcx and Pafah1b1 suggesting possible role for miRNA in regulating gene expression.

Since hyperglycemia increased neurogenesis in NSCs, we analyzed the expression of miR-124, which has been widely shown to promote neurogenesis [31]. The expression of mmu-miR-124 was increased significantly in NSCs from diabetic pregnancy (8.83±2.77 vs 1.00±0.40-folds, *p*<0.05) when compared to the control (Fig. 3D). The increased expression of miR-124 correlated with increased neurogenesis in NSCs from diabetic pregnancy (Figs. 3D and 2M).

### miRNA-mRNA Target Validation Confirms Target Prediction

**Figure 4:**
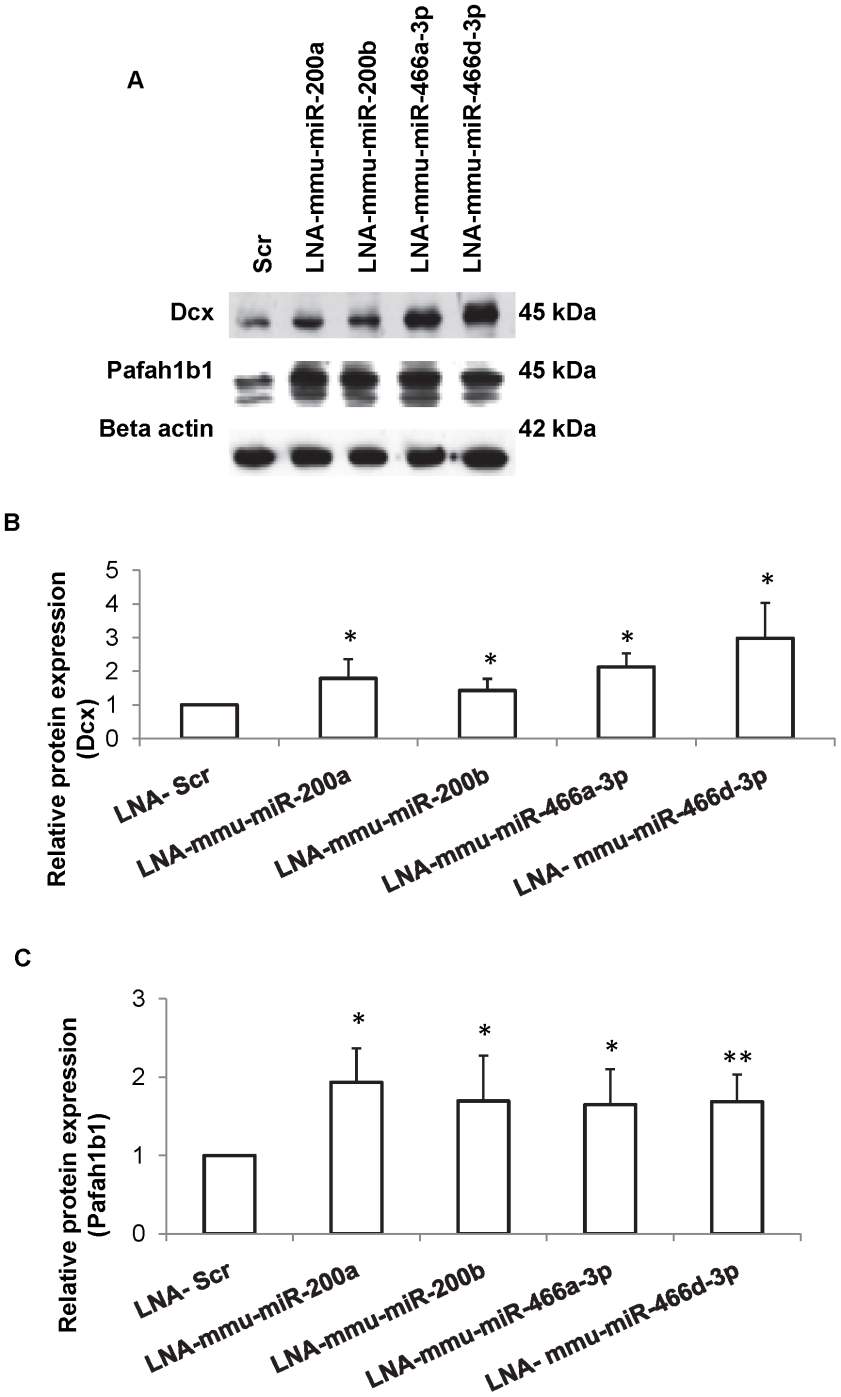
miRNA loss of function confirms Dcx and Pafah1b1 as targets. (A) Representative Western blot showing Dcx and Pafah1b1 protein expression after knockdown of specific miRNAs in NSCs. (B,C) The quantities of Dcx (B) and Pafah1b1 (C) proteins increased significantly in NSCs following knockdown of mmu-miR-200a or mmu-miR-200b or mmu-miR-466a-3p or mmu-miR-466d-3p when compared to scrambled (scr) transfected cells. Data is represented as mean ± SD from at least four independent experiments, *p<0.05. **p<0.01.

We performed miRNA loss of function study in order to validate the predicted miRNA-mRNA interactions, since the expression of selected miRNAs was significantly downregulated in NSCs from diabetic pregnancy. Individual miRNAs were knocked down in NSCs isolated from normal pregnancy using miRCURY LNA ™ miR inhibitors. The expression of the target proteins (Dcx and Pafah1b1) were analyzed by western blotting 48 h post miRNA knockdown (Fig. 4A). Knockdown of miRNAs, mmu-miR-200a, or mmu-miR-200b, or mmu-miR-466a-3p or mmu-miR-466d-3p resulted in increased expression of Dcx (1.78±0.57-folds, *p*<0.05; 1.42±0.34-folds, *p*<0.05; 2.12±0.40-folds, *p*<0.05; 2.98±1.05-folds, *p*<0.05 respectively) (Fig. 4B) and Pafah1b1 (1.93±0.43-folds, *p*<0.05; 1.69±0.57-folds, *p*<0.05; 1.64±0.45-folds, *p*<0.05; 1.68±0.34-folds, *p*<0.01 respectively) (Fig. 4C) proteins in NSCs.

### miRNA Knockdown Increases Gliogenesis and Neurogenesis in NSCs

Given that we observed increased expression of proteins involved in neurogenesis, and neuronal migration after knockdown of miRNAs in NSCs, we examined the role of these miRNAs in NSC fate determination. miRNAs, mmu-miR-200a, mmu-miR-200b, mmu-miR-466a-3p and mmu-miR-466d-3p were knocked down individually in NSCs in culture. Following knockdown of miRNAs, differentiation was induced and the neuronal and glial lineage populations were estimated by immunocytochemical analysis.

**Figure 5:**
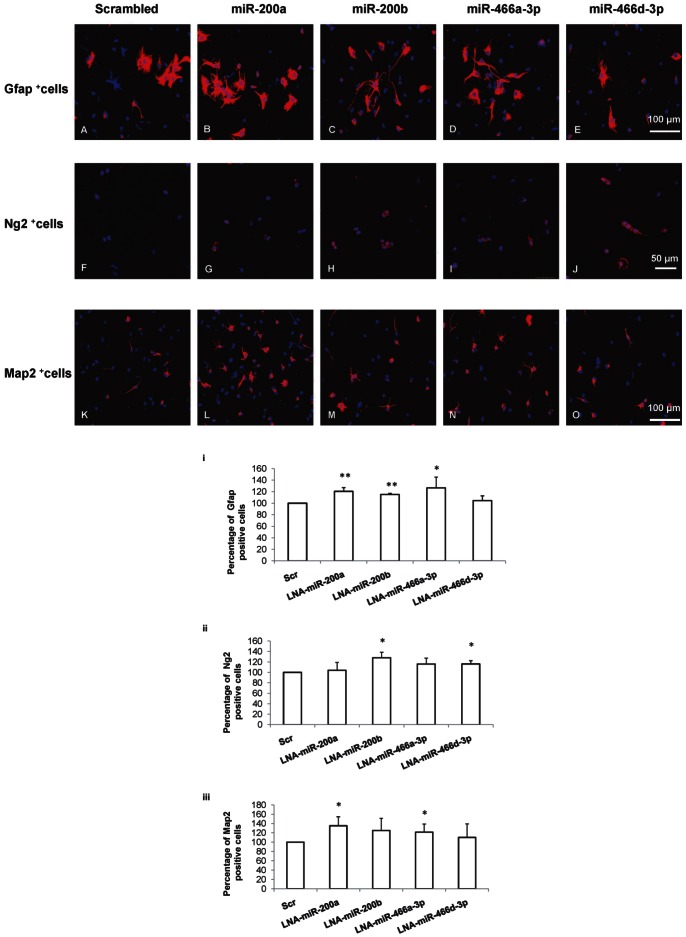
miRNAs regulate NSC fate. The effect of miRNA knockdown on NSC fate was assayed. Specific miRNAs were knocked down in NSCs and the percentage of Gfap, Ng2 or Map2 positive cells were quantitated in differentiated cells. Confocal images showing the expression of Gfap positive cells (A–E), Ng2 positive cells (F–J) and Map2 positive cells (K–O) (red) in differentiated cells following knockdown of miRNAs mmu-miR-200a or mmu-miR-200b or mmu-miR-466a-3p or mmu-miR-466d-3p in NSCs. Quantitative analysis shows the percentage of Gfap positive cells (i), Ng2 positive cells (ii) and Map2 positive cells (iii) following knockdown of specific miRNAs. Data is represented as mean ± SD from at least three independent experiments, *p<0.05. **p<0.01.

There was significantly increased astrogenesis as indicated by increased Gfap positive cells following knockdown of miRNAs, mmu-miR-200a (120.52±6.54%, *p*<0.01) or mmu-miR-200b (115±2.24%, *p*<0.01) or mmu-miR-466a-3p (126.53±18.87%, *p*<0.05) compared to scrambled (Scr) transfected cells (Fig. 5 A–E, i). Similarly, the knockdown of miRNA mmu-miR-200b (128.09±10.46%, *p*<0.05) or mmu-miR-466d (116.05±6.19%, *p*<0.05) increased the number of Ng2 positive cells when compared to scrambled transfected cells (Fig. 5F–J,ii). Knockdown of only mmu-miR-200a (135.25±19.34%, *p*<0.05) or mmu-miR-466a-3p (121.54±17.29%, *p*<0.05) significantly increased the number of Map2 positive cells compared to scrambled transfected cells (Fig. 5K–O, iii), signifying increased neurogenesis.

## Discussion

Maternal diabetes has been shown to alter the expression of genes involved in neurogenesis, neuronal migration, and differentiation of NSCs, leading to neural tube defects [7], [11]. However, the exact mechanism behind the deregulation of genes leading to brain defects is not clear. It has long been argued that epigenetic mechanisms are involved in modulating the expression patterns of multiple genes that are implicated in impaired neurodevelopment in offspring of diabetic pregnancy. Although the management of diabetes during pregnancy has reduced the occurrence of congenital malformations greatly, infants of diabetic mothers have been shown to develop neuropsychological deficits [32]. Analyzing phenotypically normal embryos may be essential in understanding the molecular details behind the occurrence of neuropsychological deficits in offspring of diabetic mothers. The present study clearly shows that hyperglycemia activates epigenetic mechanisms that result in altered gene expression in NSCs derived from diabetic pregnancy and cultured in PG medium.

Chromatin packaging is controlled by reversible histone modifications and DNA methylation patterns and determines accessibility and availability of DNA for transcription *via* specific acetylation or methylation marks on histone. Heterochromatin regions that regulate gene expression by adding or removing silencing marks to genes [33], are known to present at the nuclear periphery [34] as revealed in TEM analysis. Among the histone lysine methylation, H3K9me3 residue has been shown to be associated with gene promoters that are transcriptionally repressed and is most abundant in heterochromatin regions [35]. High glucose has been reported to alter gene expression by acetylation or methylation of lysine residues on histone H3 [20], [21]. Taken together, the increased global H3K9me3 and decreased H3K9ac observed in NSCs from diabetic pregnancy and NSCs exposed to HG *in vitro* indicate that histone modifying enzymes are possibly activated by hyperglycemia, resulting in altered gene expression. Further, global methylation assay revealed increased DNA methylation in NSCs from diabetic pregnancy and NSCs exposed to HG *in vitro*. The increased DNA methylation may be responsible for aberrant gene expression (possibly due to DNA hypermethylation at gene promoters) in NSCs from diabetic pregnancy and NSCs exposed to HG *in vitro*. Although global DNA methylation levels were found to be increased, upregulation of Pafah1b1 gene expression in NSCs from diabetic pregnancy and NSCs exposed to HG *in vitro* was not due to changes in CpG methylation.

Neurogenesis and neuronal migration are key events controlling proper development of the brain. Defective neurogenesis or neuronal migration results in brain abnormalities [36], [37]. In order to understand the role of hyperglycemia on fate specification of NSCs, we have analyzed expression of two genes (Dcx and Pafah1b1) involved in neurogenesis and neuronal migration [37]–[39]. Both Dcx and Pafah1b1 were found to be significantly altered in the brain tissue of embryos from diabetic mice by our previous microarray studies. Dcx is expressed by migrating neuronal precursors [40] and functions upstream to Pafah1b1 gene during neuronal migration [41]. Further, Pafah1b1 is required for accurate orientation of spindle (and therefore proliferation) in neuroepithelial stem cells and radial glial progenitor cells [42]. Upregulation or downregulation of Pafah1b1 or Dcx have been shown to affect brain development. Decreased expression of Dcx and/or Pafah1b1 contributes to Lissencephaly that is characterized by “smooth brain appearance” [43]. Increased expression of Dcx inhibits proliferation and promotes migration of human neural progenitors [44]. Increased expression of Pafah1b1 has been shown to affect brain development including size and cellular organization in humans and mice [45]. In the present study, an increased expression of Dcx, Pafah1b1 proteins in the NSCs from diabetic pregnancy appear to be associated with increased neurogenesis. It appears that hyperglycemia promotes neurogenesis at the cost of gliogenesis since the expression levels of Gfap and Ng2, markers of astrocytes and oligodendrocytes respectively, were found to be decreased in NSCs from diabetic pregnancy. The decreased expression of glial markers in NSCs exposed to hyperglycemia could also be due to the fact that the gliogenesis has been shown to be preceded by the neurogenesis in the developing cortex [46]. Although there was no change in the differentiation pattern in NSCs from embryos of control and diabetic pregnancy at different time points (3 days and 6 days of differentiation), the possibility of delayed gliogenesis cannot be excluded since NSCs were isolated from only one embryonic stage (E113.5) in this study. Overall, it is suggested that hyperglycemia perturbed the cell fate choice between neurons and glia. It is not clear what drives the hyperglycemia-induced glia–neuron switch, although several genes appear to be involved.

It is well known that miRNAs regulate gene expression *via* binding to 3′UTR sequences causing repression or degradation of the target mRNAs. miR-200 family has been reported to regulate olfactory neurogenesis in mouse and zebrafish models [47] and the expression of few members of miR-466 family have been shown in mouse ocular tissue [48]. This study is the first to report the expression of miRNAs mmu-miR-200a, mmu-miR-200b, mmu-miR-466a-3p and mmu-miR-466 d-3p in NSCs obtained from mouse embryonic forebrain. Further, we confirmed that Dcx and Pafah1b1 were targets of miRNAs mmu-miR-200a, mmu-miR-200b, and mmu-miR-466a-3p and mmu-miR-466d-3p by using knockdown approach. miR-124 is expressed abundantly in the mouse brain [49], specifically in the neurons and its expression is found to increase during development [50]–[52]. Several studies have demonstrated the role of miR-124 in promoting neurogenesis and neuronal differentiation [52]–[55]. In the present study, increased neurogenesis observed correlated with increased expression of neuron specific miRNA, miR-124.

Recently miRNAs have been shown to promote and regulate cell lineage specification in mouse and human cells [56]. Since knockdown of specific miRNAs increased the expression of proteins involved in neurogenesis and neuronal migration, we examined their roles in NSC fate determination. We report novel role of miRNAs mmu-miR-200a, mmu-miR-200b, mmu-miR-466a-3p and mmu-miR-466d-3p in regulating neurogenesis and gliogenesis *in vitro* by targeting and upregulating Gfap, Map2 and Ng2 proteins either through direct target or through an indirect regulation which needs detailed evaluation. Since each of the miRNAs are reported to contain hundreds of mRNA targets (miRwalk database), it is possible that many targets are involved in this complex process of NSC fate determination. Altered NSC fate due to differential miRNA expression induced by hyperglycemia may explain the basis for patterning defects observed in the developing brain exposed to maternal diabetes.

In conclusion, this study demonstrates that epigenetic mechanisms are activated in NSCs by hyperglycemia, resulting in chromatin reorganization, altered histone H3K9 status, increased global DNA methylation, and altered miRNA expression thereby promoting increased expression of neuronal and glial lineage markers. This study is clinically relevant owing to the fact that epigenetic changes are persistent even when NSCs from diabetic pregnancy were cultured in normoglycemia and NSCs from non-malformed embryos are sensitive to glucotoxicity suggesting that phenotypically normal embryos are genotypically distinct and deficient. Since epigenetic changes are reversible, using them as therapeutic targets may improve fetal outcomes in diabetic pregnancy.

## Supporting Information

Figure S1 **(A) Phase contrast image of Neural stem cells (NSCs) in culture, as free floating neurospheres.** Scale bar: 150 µm. (B–E) Confocal images of neurosphere stained with intermediate filament marker Nestin in red. DAPI is used to stain the nucleus blue. (B, C) All neurospheres obtained from control (B) or diabetic pregnancy (C) express immunoreactivity to Nestin. (D, E) Panel shows the expression of Nestin by all cells within a neurosphere from embryos of control (D) or diabetic pregnancy (E).
(TIFF)

Figure S2 **Bisulphite sequencing of DNA from NSCs from control, diabetic pregnancy and that exposed to HG** ***in vitro*** **was performed and methylation pattern was represented as lollipop grid.** There was no change in CpG methylation status at Pafah1b1 promoter in NSCs from diabetic pregnancy or that exposed to HG *in vitro* when compared to the control. Data from six clones is represented here where each row represents the sequencing information received from a single clone across 27 CpG sites (−310 to −46). Open circles represent unmethylated CpG sites and closed (shaded) circles represent methylated CpG sites.
(TIFF)

Figure S3 **(A–H) Neurospheres from embryos of control and diabetic pregnancy were allowed to differentiate for six days** ***in vitro*** **and the expression of neuronal (Map2), glial (Gfap, Ng2), and Nestin positive cell populations were determined by immunocytochemistry.**
(TIFF)

Tables S1 **Includes Tables S1, S2, S3.**
(DOCX)
