# Supplementary figures and images for: Analysis of Epigenetic Factors in Mouse Embryonic Neural Stem Cells Exposed to Hyperglycemia
Source: PLoS One. 2013 Jun 11;8(6):e65945. doi: 10.1371/journal.pone.0065945 (PMC3679101; doi:10.1371/journal.pone.0065945)

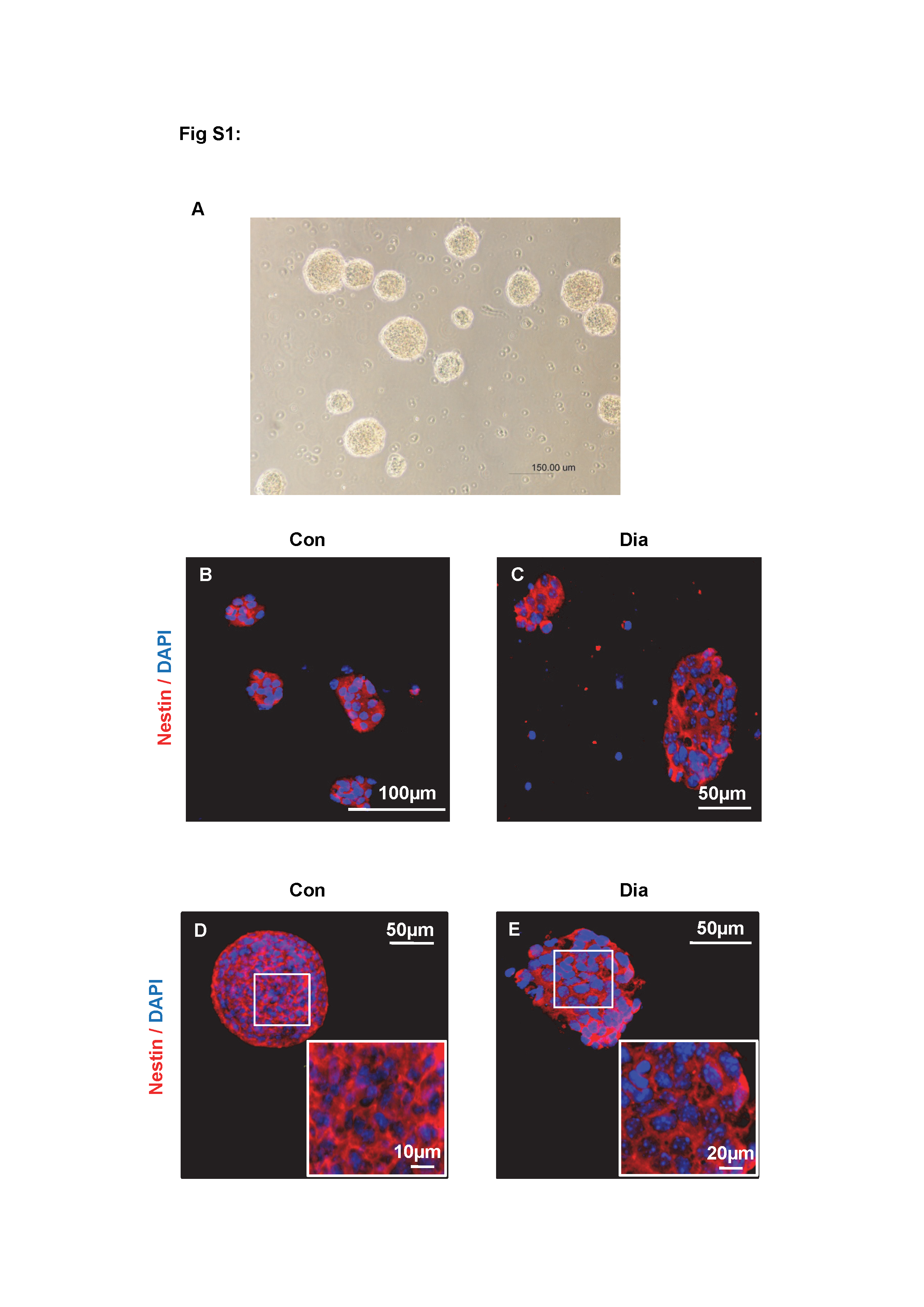

Supplement: Figure S1 — (A) Phase contrast image of Neural stem cells (NSCs) in culture, as free floating neurospheres. Scale bar: 150 µm. (B–E) Confocal images of neurosphere stained with intermediate filament marker Nestin in red. DAPI is used to stain the nucleus blue. (B, C) All neurospheres obtained from control (B) or diabetic pregnancy (C) express immunoreactivity to Nestin. (D, E) Panel shows the expression of Nestin by all cells within a neurosphere from embryos of control (D) or diabetic pregnancy (E). (TIFF) [file pone.0065945.s001.tiff]

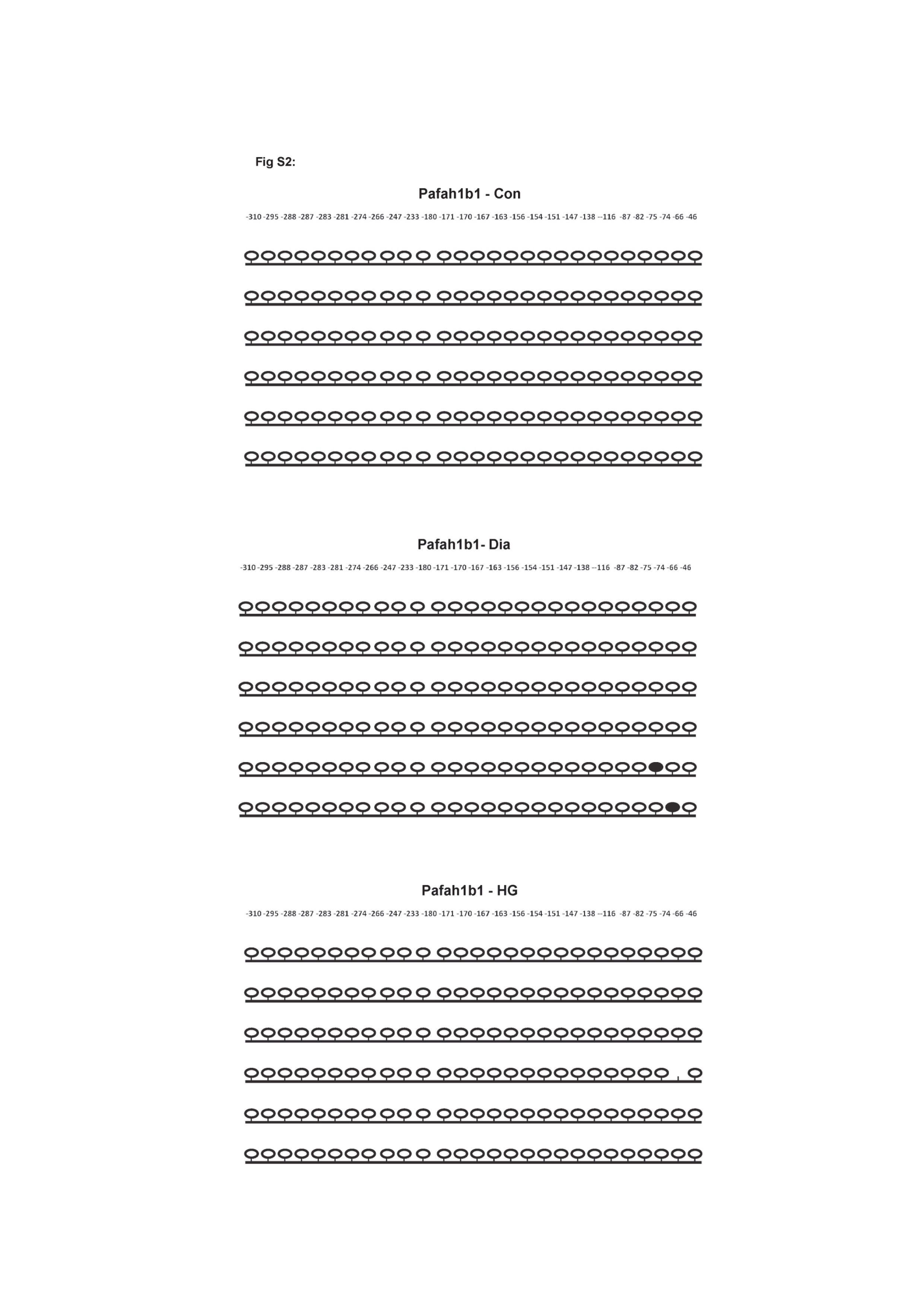

Supplement: Figure S2 — Bisulphite sequencing of DNA from NSCs from control, diabetic pregnancy and that exposed to HG in vitro was performed and methylation pattern was represented as lollipop grid. There was no change in CpG methylation status at Pafah1b1 promoter in NSCs from diabetic pregnancy or that exposed to HG in vitro when compared to the control. Data from six clones is represented here where each row represents the sequencing information received from a single clone across 27 CpG sites (−310 to −46). Open circles represent unmethylated CpG sites and closed (shaded) circles represent methylated CpG sites. (TIFF) [file pone.0065945.s002.tiff]

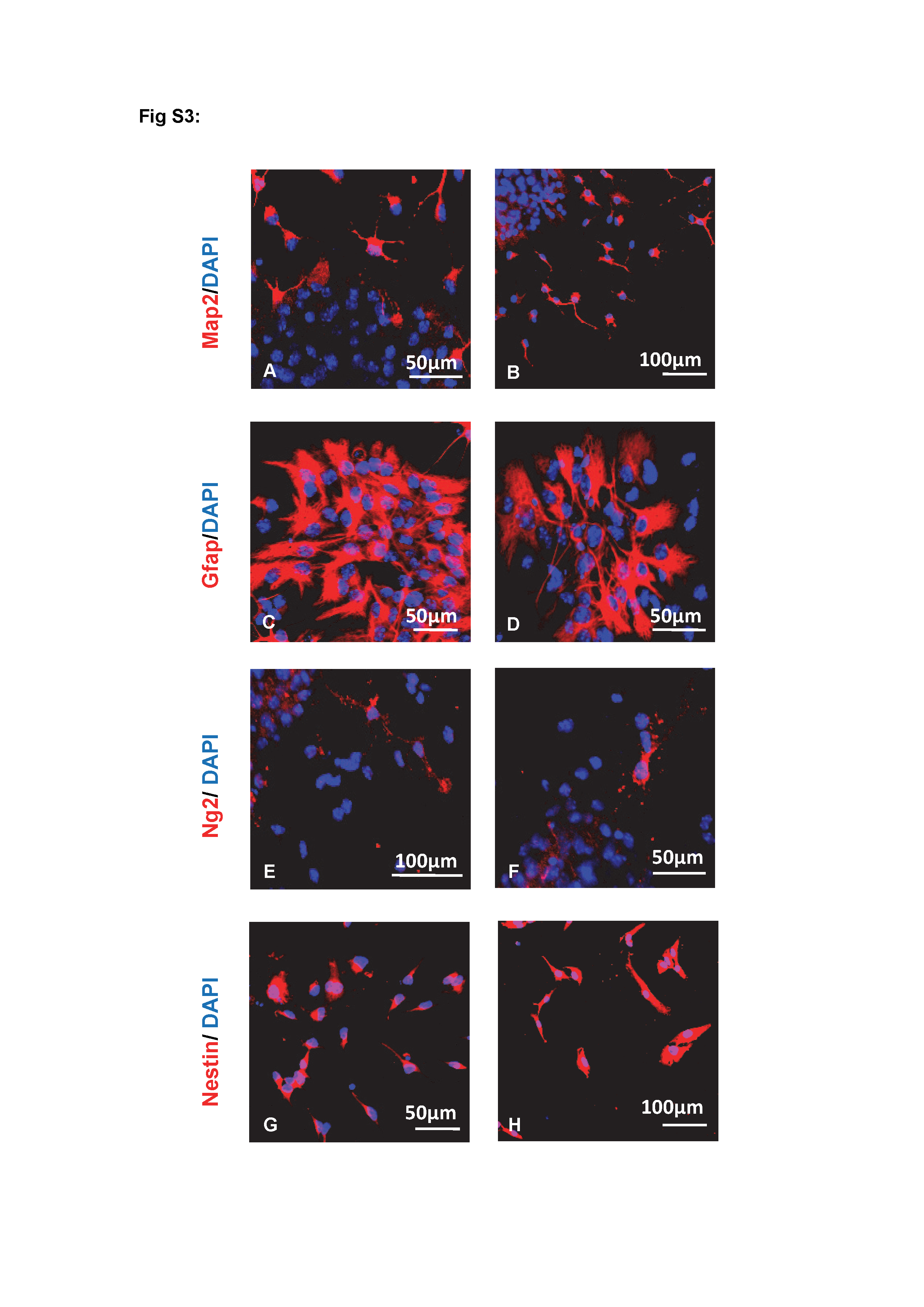

Supplement: Figure S3 — (A–H) Neurospheres from embryos of control and diabetic pregnancy were allowed to differentiate for six days in vitro and the expression of neuronal (Map2), glial (Gfap, Ng2), and Nestin positive cell populations were determined by immunocytochemistry. (TIFF) [file pone.0065945.s003.tiff]
