# Supplementary material for: Analysis of Epigenetic Factors in Mouse Embryonic Neural Stem Cells Exposed to Hyperglycemia
Source: PLoS One. 2013 Jun 11;8(6):e65945. doi: 10.1371/journal.pone.0065945 (PMC3679101; doi:10.1371/journal.pone.0065945)
Supplement: Tables S1 — Includes Tables S1, S2, S3. (DOCX) [file pone.0065945.s004.docx]

**Table S1: Primers used for DNA methylation assay**

| **Pafah 1b1 DNA** | **Forward primer** | **Reverse primer** | **Product size** |
| --- | --- | --- | --- |
| Genomic | AAAGGAAGCATCCTGGCTTGGC | GTTGGGGCAGCTCCTGTGACAG | 399 bp |
| Bisulphite modified | AAAGGAAGTATTTTGGTTTGGT | GTTGGGGTAGTTTTTGTGATAG | 399 bp |

**Table S2: Sequences of miRNA *in situ* hybridization probes**

| **miRNA inhibitor** | **Sequence (5’-3’)** |
| --- | --- |
| hsa-miR-200b(mmu-miR-200b) | TCATCATTACCAGGCAGTATTA |
| mmu-miR-466d-3p | CTATGTGTGCGTGTATGTATA |
| U6(hsa/rno/mmu) | CACGAATTTGCGTGTCATCCTT |

**Table S3: sequences of miRNA inhibitors**

| **miRNA inhibitor** | **Sequence (5’-3’)** |
| --- | --- |
| hsa-miR-200a (mmu-miR-200a) | CATCGTTACCAGACAGTGTT |
| hsa-miR-200b (mmu-miR-200b) | CATCATTACCAGGCAGTATT |
| mmu-miR-466a-3p | CTTATGTGTGCGTGTATGTATA |
| mmu-miR-466d-3p | CTATGTGTGCGTGTATGT |
| Scrambled | GTGTAACACGTCTATACGCCCA |
